# Protein and Energy Requirements for Maintenance and Growth in Juvenile Meagre *Argyrosomus regius* (Asso, 1801) (Sciaenidae)

**DOI:** 10.3390/ani11010077

**Published:** 2021-01-04

**Authors:** Ignacio Jauralde, Jorge Velazco-Vargas, Ana Tomás-Vidal, Miguel Jover Cerdá, Silvia Martínez-Llorens

**Affiliations:** 1 Research Group of Aquaculture and Biodiversity, Institute of Animal Science and Technology, Universitat Politècnica de València, Camino de Vera 14, 46071 València, Spain; atomasv@dca.upv.es (A.T.-V.); mjover@dca.upv.es (M.J.C.); silmarll@dca.upv.es (S.M.-L.); 2 Faculty of Environmental Science, Pontifical University Catholic of Ecuador in Esmeraldas (PUCESE), C/Espejo y Santa Cruz S/N, Esmeraldas 080150, Ecuador; jvelazco_47@hotmail.com

**Keywords:** *Argyrosomus regius*, nutritional requirement, maintenance requirement

## Abstract

**Simple Summary:**

The meagre is a fish species of recent interest in aquaculture, because of its fast growth and flesh quality. Nevertheless, it hasn’t been studied enough, and feed producers do not have enough information about the nutrient requirements to optimize the feed diets of the meagre. This study measures the growth response of this fish to several amounts of food and gives information about the proportion of protein and energy that should be included in its diet, as well as the recommended amount of food to optimize its growth.

**Abstract:**

The meagre is a carnivorous species and might be a suitable candidate species for the diversification of aquaculture in the Mediterranean region. This is based on its high growth and flesh quality. Nevertheless, there is little information available about its growth rates and nutrient requirements. The objective of this study was to determine the protein and energy requirements of juvenile meagre (*Argyrosomus regius*). Two trials for different weights of 53 and 188 g were conducted with rations from starvation to apparent satiation with the scope of studying its nutritional needs. In the first trial, the initial mean body weight of the fish was 53 g, and they were fed at feeding rates, measured as a percentage of the body weight, of 0, 0.75, 1.5, 2.5, 3.5, and 4.5%, with two replicates per treatment. In a second trial, another group with approximately 188 g of initial body weight was fed at feeding rates of 0, 0.5, 1.5, and 2.5%, with two replicates per treatment. The optimum thermal growth coefficient was obtained with a feed intake of 2.2% day^−1^ in trial A and 1.73% day^−1^ in trial B. The digestible protein (DP) intake for maintenance was determined as 0.57 g kg^−0.7^ day^−1^, the DP intake for maximum growth was 6.0 g kg^−0.7^ day^−1^, and the point for maximum efficiency in protein retention was 1.8 g kg^−0.7^ day^−1^. The requirement for digestible energy (DE) intake for maintenance was recorded at 25.4 kJ kg^−0.82^ day^−1^, the DE intake to maximize growth was 365 kJ kg^−0.82^ day^−1^, and the point for maximum efficiency in energy retention occurs with a digestible energy intake of 93 kJ kg^−0.82^ day^−1^. The requirements and retention efficiency of protein and energy in *Argyrosomus regius* tend to be within the range other fish species. The maintenance needs are in agreement with species with low voluntary activity and growth requirements in agreement with fast-growth species.

## 1. Introduction

The meagre (*Argyrosomus regius*) is an emerging species with 15,000 tons of production in the Mediterranean region [1,2,3,4] given its high growth rate and flesh quality. As body and fillet traits of meagre have shown a very high dressing content with a negligible amount of mesenteric and muscular fat compared to other cultured fish, this species becomes even more interesting for industrial processing and human consumption purposes [5]. Its production has increased to 15,000 tons per year, when ten years ago it was less than 1000 (FAO, Rome, Italy).

The on-growing techniques used until now are based on the rearing of gilthead sea bream (*Sparus aurata*) and European sea bass (*Dicentrarchus labrax*), but between meagre and sea bass or sea bream there are some important differences: The meagre’s high growth rate enables them to reach 1 kg in 10 months. Usual commercial weight ranges from 1.5 to 3 kg [6,7,8]. Chatzifotis et al. [9] obtained a specific growth rate (SGR) ranging between 0.7 and 1.3% day^−1^ for fish with final weights between 46 and 72 g; if this data referred to thermal growth coefficient (TGC), these values would range between 1.3 × 10^−3^ and 1.98 × 10^−3^. The highest growth was obtained with the highest dietary protein level studied (50% crude protein (CP)) and Martínez-Llorens et al. [7] found that fish fed with a commercial diet with 47% of crude protein and 20% of crude lipid showed the best results in meagre growth. Nevertheless, information on its growth rates and nutrient requirements are scarce.

The meagre is a carnivorous species known to show low activity in cages and, in its natural environment, it feeds on mysidacea, decapoda, and teleostei [9]. The dietary protein level needed for maximum growth of carnivorous species under culture conditions varies between 40% and 55% [10]. Generally, the increase in dietary protein can lead to improved fish production and brood stock, especially for carnivorous fish [11,12,13,14]. However, excess protein supplied in feeds is metabolized as an energy source and increases production of nitrogenous waste material that is excreted in the water, which may be detrimental to fish growth and culture environment [14,15].

Protein is usually a fairly constant component in bodyweight in fish species. In general, fish require a higher level of dietary protein than terrestrial farmed vertebrates [16]. The protein requirements vary between species, with carnivorous fish generally having higher dietary protein requirements than omnivorous and herbivorous species [17]. In meagre the best growth results are obtained with a dietary protein level of 50% or higher [9], and maximum growth can be achieved with diets with low levels of lipids, as it is a carnivorous species with a high protein requirement in comparison to other species commonly cultured in the Mediterranean Sea, such as European sea bass or gilthead sea bream, but others studies evidenced the relative importance of diet composition and how growth is mainly influenced for a correct digestible protein/digestible energy (DP/DE) ratio [18,19,20,21,22]. For these two species, dietary protein inclusion is commonly around 45%. Meagre juveniles seems to have similar optimum dietary lipid levels to other Mediterranean species, and dietary lipid level excess should be avoided because an increase from 17% to 21% resulted in higher fat accumulation and impaired growth performance [1,9]. The study by Martínez-Llorens et al. [7] found that fish fed with a commercial diet with 47% crude protein and 20% crude lipid showed the best results in meagre growth. In most studies it seems that the highest protein/lipid level studied obtained the best growths results.

These studies determined the best protein and lipid levels of the diet for *A. regius* based on percentage levels in the diet, but it is still necessary to specifically study its nutritional requirements. Nutritional requirements are not expressed as a percentage of the diet, but rather in terms of absolute daily feed requirements per unit of weight and weight gain and, once the nutritional requirements are known, the diet percentages of protein and energy can be determined attending to body composition and growth rate [18,19,20].

Factorial models have been used to estimate energy and protein requirements in growing fish. The factorial model used in past studies can be expressed as:

$$Requirement=M\times BW\left(Kg^{b}\right)+G\times 1/k$$

where the requirement of a nutrient is expressed as function of a maintenance value *M*, body weight (*BW*) is expressed in metabolic weight with exponent *b*, *G* is the expected retention of that nutrient in the fish, and *k* is the net efficiency of that nutrient.

Maintenance and efficiency can be determined by dose-response trials to increment ranges of feeding rate of a diet [19].

Usually, both digestible protein (DP) and digestible energy (DE) intake are used to estimate maintenance requirements and efficiency of retention (*k*). This kind of trial has been performed on *S. aurata* [19,20,21], *D. labrax* [21,22,23], *Epinephelus aeneus* [21,24], *Argyrosomus japonicus* [25], *Sciaenops ocellatus* [26], *Oncorhynchus mykiss* [27,28,29], *Bidyanus bidyanus* [30], *Gadus morhua* [31], *Lates calcarifer* [32], and *Salmo salar* [33].

The response curve to increasing levels of DP intake has been described as both linear [20,23,24,34] or curvilinear [19,25,35] and in fish, this pattern of protein deposition may vary considerably depending on species, diet, and experimental conditions (e.g., initial weight and temperature).

The objectives of this study were to determine protein and energy requirements, protein and energy retention efficiencies, and maintenance requirements of juvenile *Argyrosomus regius*.

## 2. Materials and Methods

The trial was conducted in eight octagonal concrete tanks (4000 L) inside a marine water recirculation system (65 m^3^ of capacity) with a rotary mechanic filter and a gravity biofilter with a capacity of around 6 m^3^ at the aquaculture laboratory of the Animal Science Department at Polytechnic University of Valencia (Valencia, Spain). All tanks were equipped with three cages of 1000 liters or two cages of 1500 liters (Table 1) and aeration, and water was heated by a heat pump installed in the system. The equipment used to control water parameters was an oxy-meter (OxyGuard, Handy Polaris V 1.26, Farum, Denmark), a refractometer with a 0–100 g L^−1^ range (Zuzi, A67410, Beriain, Spain), and a kit applying the colorimetric method to determine nitrate, ammonia, and nitrite concentrations. The kits were obtained from AquaMerck (Merck KGaA, Darmstadt, Germany). During the trial, water temperature (19 ± 1 °C) and dissolved oxygen (7.36 ± 0.4 mg L^−1^) were measured on a daily basis. Salinity (27 ± 1 g L^−1^), pH (7.3 ± 0.5), NH_4_^+^ (0.0 mg L^−1^), NO_2_^−^ (0.22 ± 0.2 mg L^−1^), and NO_3_^−^ (46.1 ± 3.7 mg L^−1^) were measured three times a week. The photoperiod was natural and all tanks had similar light conditions.

Two trials were conducted at Polytechnic University of Valencia. The fish in the first trial were supplied by IRTA (San Carles de la Rápita, Tarragona, Spain), with an initial body weight of 53 g. The fish in the second trial had an initial body weight of approximately 188 g and were supplied by IFAPA Center “El Toruño” (Santa María Port, Cádiz, Spain). All fish were acclimatized to the experimental conditions along a 30-day period, and were fed a commercial diet (47% crude protein (CP), 20% crude lipid (CL), 5.8% ash, and 1.5% crude fiber (CF), Skretting, Burgos Spain). The two trials (A and B) are summarized in Table 1.

All fish were weighed approximately every 4 weeks. Prior to weighing, the fish spent 24 h under starvation and for the weigh-in, the fish were anaesthetized with 30 mg L^−1^ of clove oil (Guinama^®^, Valencia, Spain) containing 87% eugenol.

With the aim of determining the body composition and retentions, 5 fish per trial were sacrificed at the beginning of it to determine their body composition and, at the end of the trial, 10 fish per cage were randomly collected for the analysis.

The diet used in both trials and during the digestibility analysis period was a commercial diet (Skretting, Burgos, Spain). Chemical analyses were performed at the Food Laboratory of Polytechnic University of Valencia, and diet composition is described in Table 2.

In all treatments (Table 1), the fish were fed from Monday through Friday twice a day, and just once on Saturdays. During feeding, observers checked that all of the feed offered was eaten by the fish, ensuring equal distribution of the pellets among the fish. For the first meal of the day, the entire ration was given and, if the fish showed a lack of appetite, feeding was stopped and any remains were given in the second meal. If the fish displayed a lack of appetite during the second meal, then feeding was stopped, the remaining food was weighed, and the feeding rate (FR) was corrected from the theoretical FR to the actual FR.

The apparent digestibility experiment was carried out in the same tanks at the end of the growth experiment. Two parallel trials were performed: one with fish weighing 55 g and another one when fish weight was at 120 g. All fish were fed to satiety and fecal collection took place 15 h later (09:00). Extraction was achieved by means of stripping (applying pressure on the ventral region, from the pelvic fins to the anus). Wet fecal content was collected and dried at 60 °C for 48 h before analysis (CP, energy, and acid insoluble ash (AIA) were used to calculate the apparent digestibility coefficient, ADC).

Digestibility coefficients of energy and protein were determined by fecal analysis with the following formula:

ADC(%)=100×[1−(marker in diet/marker in faeces)×(N in faeces/N in diet)]

where *N* is the nutrient.

The composition of diet, fish carcasses, and feces was analyzed following AOAC (1990) procedures: dry matter (105 °C to constant weight), ash (incinerated at 550 °C to constant weight), CP (*N* × 6.25) by the Kjeldahl method after acid digestion (Kjeltec 2300 Auto Analyser; Tecator, Höganas, Sweden), and CL extracted with diethyl ether (Soxtec 1043 extraction unit; Tecator). The energy of feed and feces were determined by direct combustion in an adiabatic bomb calorimeter (Parr Model 1108 oxygen combustion bomb; Illinois, IL, USA). All analyses were performed in triplicate except for fecal analysis, which was performed in duplicate. The AIA content of feeds and feces was estimated by the method suggested by Atkinson et al. [36].

Growth data and feed utilization were treated using analysis of variance (ANOVA factorial, initial weight was used as a covariate) [37]. The Newman–Keuls test was used to assess multiple comparison tests, and the confidence interval was set at 95% (Stat graphics, Statistical Graphics System, Version Plus 5.1, Herndon, VA, USA).

Quadratic regression analyses were applied, where the thermal-unit growth coefficient (TGC) was a function of feed intake using the expression:

$$y=a+bx+cx^{2}$$

Optimum feed intake was obtained by deriving this equation and equalizing it to zero.

The equation used to describe the response retention curves was:

$$y=\;a\times \left[1-e^{-b\left(x-c\right)}\right]$$

where *y* is protein retention (PR) or energy retention (ER), *x* is the digestible protein (DP) intake or digestible energy (DE) intake, a is the plateau value for the curve, b is the constant characterizing the steepness of the curve, and c is the DP intake or DE intake at *y* = 0, and by definition, represents the intake for maintenance. The DP or DE intake for maximum retention is defined by the point on the abscissa representing 95% of the value of the upper asymptote on the ordinate.

Retention efficiency (gross and net efficiency) can be defined as:

Gross Efficiency= y/x

Net Efficiency = y/(x−c)

where *y* is the protein or energy retention and *x* is the DP intake or DE intake developing:

$$Gross\;Efficiency\;=\;a\times \left[1-e^{-b\left(x-c\right)}\right]/\left(x-c\right)$$

The maximum efficiency point can be calculated in two ways: graphically, as the tangent point between the retention curve and a tangent line crossing the point of origin of the coordinates, or algebraically, as the maximum point of the gross efficiency curve.

The animal study protocol was reviewed and approved by the Universitat Politècnica de València Ethical Committee. All experiments were conducted in an accredited animal care facility (code: ES462500001091) in accordance with the guidelines and regulations set forth in Directive 2010/63/EU EEC and the Spanish Royal Decree 53/2013 on the protection of animals used for scientific purposes [38].

## 3. Results

### 3.1. Growth

In both trials, after the growth period of 53 days, the final body weight and the TGC varied according to the different feeding rates. Table 3 shows the statistical results of growth and nutritional parameters of the two trials.

**Figure 1:**
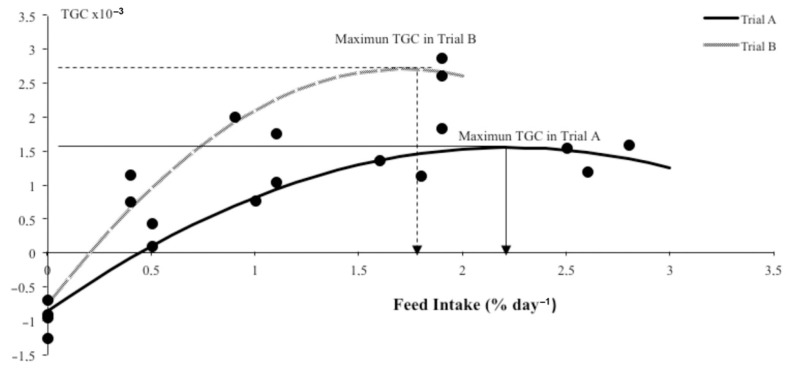
Thermal growth coefficient (TGC) response to increasing levels of feed intake (FI) and response curve of quadratic models considering trial A and trial B in meagre.

All the fish in starvation lost weight, resulting in the lowest body weight in both trials: 38 and 111 g in trials A and B, respectively. Among non-starvation rates, in trial A, the final weight of fish fed at a feeding rate of 0.75% was the lowest (57 g), and the meagre fed at the highest feeding rate (4.5%) obtained the highest final body weight (81 g), although there were no significant differences in theorical feeding rates between 1.5 and 4.5%. Similarly, in trial B, the meagre fed at 1.5 and 2.5% were significantly heavier (295 g and 339 g, respectively) than those fed at 0.5% (248 g). As Figure 1 shows, similar results were observed with regard to TGC values, and starved fish presented a negative TGC (−0.93 × 10^−3^ in trial A and −0.98 × 10^−3^ in trial B). In trial A, fish fed at 0.75% obtained the lowest TGC (0.27 × 10^−3^), and there were also no significant differences observed among fish fed at rates of 2.5, 3.5, and 4.5% (TGC of 1.25 × 10^−3^, 1.69 × 10^−3^, and 1.4 × 10^−3^, respectively). Concerning TGC in trial B, there were significant differences among feeding rates, and the TGC values increased significantly as the feeding rates increased.

In both trials, the feed intake (FI) was significantly different within a trial, and it increased as the designed feeding rate increased, as shown in Table 3.

With the aim of determining the FI to maximize fish growth (Figure 1), a second-order polynomial regression analysis was conducted, and the equation that described the relationship between TGC and the FI was as follows:

Trial A

(1) $$\mathrm{TGC}\;=\;-0.487641\;\times \;10^{-3}\times {\;\mathrm{FI}}^{2}\;+\;2.17334\;\times \;10^{-3}\times \mathrm{FI}\;-\;0.87187\;\times \;10^{-3}\;r_{\mathrm{adj}}^{2}=95\%$$

Trial B

(2) $$\mathrm{TGC}\;=\;-1.17882\times \;10^{-3}\times {\mathrm{FI}}^{2}\;+\;4.05533\;\times \;10^{-3}\times \mathrm{FI}\;-0.78587\;\times \;10^{-3}\;r_{\mathrm{adj}}^{2}\;=\;94\%$$

Optimum daily FI for maximum TGC (2.2% day^−1^ in trial A, 1.73% day^−1^ in trial B) was obtained by deriving these equations and equalizing them to zero.

### 3.2. Energy and Protein Intake

DP intake and DE intake increased significantly as feeding rates increased (Table 3). The highest value in trial A was obtained with a feeding rate of 4.5% (9.21 g DP kg^−0.7^ day^−1^ and 503.9 KJ DE kg^−0.82^ day^−1^, respectively), and, in trial B, the rate was obtained with a feeding rate of 2.5% (8.30g DP kg^−0.70^ day^−1^ and 402.3 KJ DE kg^−0.82^ day^−1^, respectively).

In both trials, the increased intake in either digestible protein or digestible energy produced a significant increase in the retention of energy and protein for the feeding rates of 0, 0.75, and 1.5, but without statistical differences among higher feeding rates (2.5, 3.5, and 4.5%) (Table 3).

### 3.3. Body Composition

Table 3 also shows the statistical results of body composition. The percentage of moisture and ash in the starved fish was the highest (Table 3), and fish under starvation also show the lowest percentages and values of protein, lipid, and energy. In trial A, the moisture, lipid, and energy contents were significantly higher at rates of 1.5, 2.5, 3.5, and 4.5% than in the fish fed at 0.75% (77.5%, 4.21%, and 5.22%, respectively); the percentage of ash did not show significant differences with regard to feeding rates, whereas protein content significantly increased feeding rate from 14.82 to 16.02. Trial B did not show significant differences in the statistical results of body composition.

### 3.4. Retention

**Figure 2:**
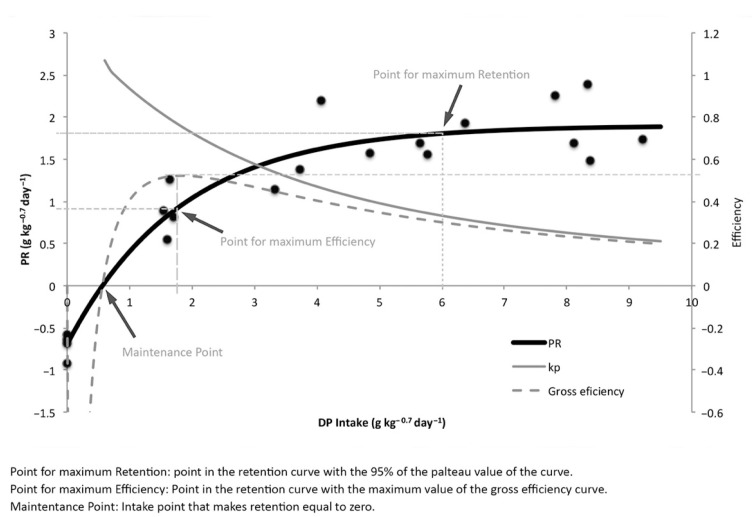
Effect of digestible protein intake (g kg^−0.7^ day^−1^) on protein retention (g kg^−0.7^ day^−1^).

**Figure 3:**
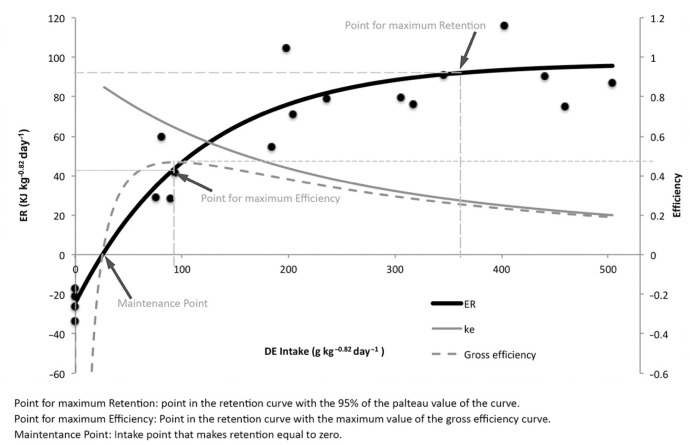
Effect of digestible energy intake (kJ kg^−0.82^ day^−1^) on energy retention (kJ kg^−0.82^ day^−1^).

The response curves to graded levels of DP intake and DE intake are essential to understanding the DP and DE requirements (Figure 2 and Figure 3). Following the usual procedure, the data are expressed in metabolic weight [19]. The asymptotic equations describing the response curve were:

(3) $$\mathrm{Protein}\;\mathrm{retention}=1.9\left(1-e^{-0.55\left(\mathrm{DP}\;\mathrm{intake}-0.57\right)}\right)\;r_{\mathrm{adj}}^{2}=\;91.5\%$$

(4) $$\mathrm{Energy}\;\mathrm{retention}=\;97.08\left(1-e^{(-0.0088\left(\mathrm{DE}\;\mathrm{intake}-25.42\right)}\right)\;r_{\mathrm{adj}}^{2}=89.1\%$$

The points for maintenance, maximum efficiency, and maximum retention were determined according to the regression models (Equations (3) and (4)) and their plots (Figure 2 and Figure 3), and are presented in Table 4 and Table 5 for both protein and energy retention. Net efficiency is included in Table 4 and Table 5. The DP intake requirement for maintenance was obtained for protein retention 0 (y = 0), and the DP intake for the maintenance point was 0.57 g DP kg^−0.7^ day^−1^. The maximum retention point was calculated at 95% of the asymptotic value. The DP intake to maximize retention was 6 g DP kg^−0.7^ day^−1^ and its associated retention at that point was 1.81 g kg^−0.7^ day^−1^, obtaining a net efficiency of protein retention of 0.33. The point of maximum efficiency was reached with a level DP intake of 1.8 g kg^−0.7^ day^−1^, and this intake produced a protein retention of 0.94 g kg^−0.7^ day^−1^, with a maximum net efficiency of 0.76.

The DE intake for the maintenance point was 25.4 kJ DE kg^−0.82^ day^−1^. The maximum retention point was obtained with a DE intake of 365 kJ DE kg^−0.82^ day^−1^, and its corresponding energy retention at that intake was 92.2 kJ kg^−0.82^ day^−1^, obtaining an energy retention net efficiency of 0.27. The point of maximum efficiency was obtained with a DE intake of 93 kJ DE kg^−0.82^ day^−1^ DE producing an ER of 43.5 kJ kg^−0.82^ days^−1^ and with a maximum net efficiency of 0.64.

## 4. Discussion

One of the goals of aquaculture production is an overall cost-effectiveness with a minimum of waste outputs. To achieve this aim, it is important to optimize feeding strategies by evaluating the effect of diet ration level over fish growth. In this sense, the curvilinear response, with a predetermined diet (DP:DE ratio), allows for the identification of the optimal ration level that maximizes fish growth and feeding efficiency [25]. In the results of this study, the TGC response curve for increasing levels of FI allow us to study the FCR associated with the TGC for two important points: the TGC that produce the best FCR, and the FCR for the maximum TGC. the results of this study showed how in the TGC response curve for increasing levels of FR (Figure 1), the maximum TGC was reached at higher FR in fingerlings than in juveniles. This data agreed with the expected results, as small fish have higher metabolic activity, i.e., they need a higher feeding rate and higher protein and energy intake.

Usually maximum TGC values for one species growing under similar farming conditions can be considered constant across a wide range of weight classes (<20 g, 20–500 g, and > 500 g in rainbow trout) and temperatures [40,41]. Mayer et al. [42] also showed some differences in TGC value for *Sparus aurata* in fish over and below 173 g, but constant inside the 5–173 g range and 173–400 g range. In the current study on meagre, the changes in TGC values from below maximum growth to maximum growth, as result of the variation of the feeding rate, was studied. The polynomial regression showed that optimum daily FI for maximum TGC (1.55 × 10^−3^) was obtained close to the highest FI, at 2.2% day^−1^ FI in trial A and at 1.73% day^−1^ (2.70 × 10^−3^) in trial B. Panettieri et al. [43] found a similar maximum TGC, recalculating from raw data, of 2.42 × 10^−3^ in meagre. Under the same experimental conditions (facilities, temperature, photoperiod), the TGC value in fingerlings was usually equal or higher than in juveniles, which was not observed in the present study. The main reason for this could be attributed to the fact that the fish in this experiment came from different batches: The fish in trial B were a better batch (higher growth potential) and adapted better to experimental conditions than the fish in trial A. Also, meagre’s standards for growth performance have not yet been established, and differences between genetic lines could be important. This opens the door to extensive aquaculture research on genetic improvement, which, together with feed optimization, could lead to high growth and high feed efficiency [44].

In mulloway (*Argyrosomus japonicus*), Pirozzi et al. [25] found that in fish of 40 g, the effect of the ration on weight, protein, and energy retention varied significantly depending on the temperature. Likewise, in fish of 127 g, the ration level—but not the temperature—affected weight, protein, and energy retention. TGC was recalculated using the weight data presented here. Mulloway of 40 g presented a TGC of 1.79 × 10^−3^ (20 °C). These results were higher than the TGC obtained in the present experiment for meagre fingerlings, and TGC in large mulloway was 1.19 × 10^−3^ (20 °C), lower than the TGC obtained in meagre juveniles (Table 3), which shows the great variability of TGC that can be expected in this fish species.

The protein and energy retention response curve to the increasing levels of the digestible nutrients proved to be very interesting for the study of the requirements. In this study, as expected and in agreement with Watanabe et al. [39] and Pirozzi et al. [25], the increase in DP and DE intake levels produced increasing retentions in meagre and other species when intakes were low, and described a plateau curve when intakes were high (Figure 2 and Figure 3). In the case of fish under starvation conditions these values were negative [18,19,21,32].

The energy and protein retention curves were independent from diet and weight in a large number of species when expressed in metabolic weight [18,19,21,32], but they were still dependent on temperature, genetics, and other factors.

To describe needs, researchers normally use the metabolic body weight of each species; for gilthead sea bream, Lupatsch et al. [20] determined the protein (kg^−0.7^) and energy requirement (kg^−0.83^) and later corrected to (kg^−0.82^). Similar results were reported by Pirozzi et al. [25] for *A. japonicus*. In this study, values of 0.7 and 0.82 were used as the theoretical metabolic exponent for body weight for *A. regius*. Protein and energy needs for maintenance in meagre were 0.57 g DP kg^−0.7^ fish^−1^ day^−1^, and 25.4 kJ DE kg^−0.82^ fish^−1^ day^−1^ (Table 4), respectively, whereas digestible protein intake requirements for maximum retention were 6.0 g DP kg^−0.7^ fish^−1^ day^−1^ and energy need for maximum growth was 365 kJ DE kg^−0.82^ fish^−1^ day^−1^.

The maintenance requirement for DP in several fish species has been recorded to be between 0.45 and 3.1 g DP kg^−0.7^ fish^−1^ day^−1^ (Table 5) [19,20,21,22,23,25,26,45], whereas the DE maintenance requirements for fish have been shown to range from 34 to 97 kJ DE kg^−0.82^ fish^−1^ day^−1^ (Table 5) and vary depending on temperature, species, and fish size. In meagre, the protein needs for maintenance was within this range and very close to the value found for *A. japonicus*. On the other hand, the energy needs for maintenance were probably the lowest found due to several reasons: The digestible energy requirements for maintenance were affected by temperature [21], and the current data had the lowest temperatures studied (comparison shown in Table 5). Overall, meagre is known for its low activity in cages, which could indicate a low standard metabolic rate and would explain the low maintenance needs.

The requirements for maximum growth of digestible energy or protein were strongly influenced by the maximum growth reached: high growth implied high retention. On the other hand, if the focus were set on net efficiencies, the results of present study would show similar values to those achieved for *Sparus aurata* [19].

Retention efficiency was included in Table 4 and Table 5 to allow for comparison with other research trials. Net protein retention efficiency was determined at 0.52 [46] and 0.64 [23] in *D. labrax*, 0.54 for *E. aeneus* [24], 0.49–0.51 for *L. calcarifer* [32], and 0.58 for *A. japonicus* [25]. Many factors affected net efficiency of the digestible protein, including the quantity and quality of dietary protein (i.e., amino-acid profile), body weight and age of fish, feed intake, and numerous environmental factors [25,47,48,49,50].

The energy retention efficiency results of the present study agreed with the ones reported by Bureau et al. [35] for fish species within the 0.4–0.7 range. These results were also similar to those reported for *D. labrax* [46], *O. mykiss* [51], and *A. japonicus* [25].

The requirements of digestible protein and energy should be studied for a specific TGC because the relation of DP/DE is extremely important to enhancing efficiency. According to several authors [13,26,52], the inclusion of inadequate quantities of protein and energy can cause a reduction in growth, and an excessive quantity of energy can also reduce feed consumption, which would lead to growth reduction as well. Besides, protein is more expensive than lipids and carbohydrates, and fish use it for tissue synthesis and growth. In addition, a decrease in DP/DE ratios has indeed shown to be extremely efficient in improving protein utilization and decreasing nitrogen losses in most farmed fish [13]. Kaushik and Seiliez [16] also indicated an optimization of the ratio between digestible protein and digestible energy through dietary digestible protein level reduction with or without a concomitant increase in the dietary non-protein digestible energy supply. The data from Table 4 can be used to estimate the optimum DP/DE ratio for the maintenance point, maximum retention, and maximum efficiency. The results for these three different feed situations are, respectively, 22.4, 16.4, and 19.5 g/MJ. As consequence, for growth the diets are recommended to have between 16.4 and 19.5 g/MJ, and diets for maintenance must contain a DP/DE ratio close to 22.4 g/MJ.

Concerning body composition, a loss of energy and protein, as well as an increase in ash content, were reported only in fish in starvation as a natural consequence. The body composition at the remaining feeding rates was similar, and only fish fed 0.75% in Trial A obtained the lowest value. It seems that 0.75% was too low for feeding fingerlings and, as previously mentioned, a higher feeding rate is required. At the same time, in Trial A the crude protein was higher in fish fed 3.5%, higher even than those fed 4.5%. Although the crude protein content of fish is kept relatively constant through its life stages and is only slightly affected by dietary factors (provided the dietary essential amino acid is adequate), the lipid content of fish is variable depending on energy intake and growth [35,53]. In general, the CP was 16% and 7% for lipids. These results are proof of the excellent meat quality of the meagre, its low fat content being its main characteristic and representing an important parameter of quality for the consumer [5].

## 5. Conclusions

The optimum TGC was obtained with values close to the highest feed intake, i.e., 2.2% in trial A and 1.73% in trial B. The DP intake for maintenance and maximum growth was recorded at 0.57 g and 6.0 g kg^−0.7^ fish^−1^ day^−1^, respectively. The optimum intake of digestible protein was 1.8 g kg^−0.7^ fish^−1^ day^−1^ for maximum protein efficiency retention. The DE intake requirements for maintenance and growth maximization were 25.4 kJ kg^−0.82^ fish^−1^ day^−1^ and 365 kJ kg^−0.82^ fish^−1^ day^−1^, respectively, and the optimum point for maximum energy efficiency was 93 kJ kg^−0.82^ fish^−1^ day^−1^. The retention efficiency of protein and energy in *Argyrosomus regius* tends to be within the range reported for other fish species.

The range of values for net protein efficiency found in the present study shows the potential of this species, which is able to either reach high efficiency or achieve growth at a high speed with lower efficiency. The future challenge is to achieve high efficiency as well as high growth by determining the ideal amino acid profile for maximum growth and the correct DP/DE ratio in the diet based on protein and energy requirements.

## Figures and Tables

**Table 1:** Experimental design.

Parameters	Trial A	Trial B
Initial weight	53 g	188 g
Number of fish	169	77
Experimental system	12 cages of 1000 liters in 4 tanks of 4000 L, recirculating saltwater system	8 cages of 1500 liters in 4 tanks of 4000 L, recirculating saltwater system
Feeding rate	0, 0.75, 1.5, 2.5, 3.5, and 4.5%	0, 0.5, 1.5, and 2.5%
Feeding	1–2 times daily (hand feeding)	1–2 times daily (hand feeding)
Replicates	2	2
Duration	53 days	53 days

**Table 2:** Diet composition and proximate analysis.

Commercial Diet	
Dry matter (%)	93
Crude protein (%)	46.06
Crude lipid (%)	19.46
Ash (%)	6.35
Crude fibre (%)	1.5
NFE ^1^ (%)	26.63
GE ^2^ (MJ kg^−1^)	24.26
CP/GE (g MJ^−1^)	18.98
CDMs ^3^(%)	64.6
CDP ^4^(%)	84.85
CDE ^5^ (%)	84.94
DP ^6^ (g kg^−1^)	39.08
DE ^7^ (MJ kg^−1^)	20.60

^1^ NFE (nitrogen-free extract) calculated = 100-%CP-%CL-%Ash-%CF.; ^2^ GE = gross energy, determined by direct combustion in an adiabatic bomb calorimeter; ^3^ CDMs = apparent digestibility coefficients for dry matter; ^4^ CDP = apparent digestibility coefficients for crude protein; ^5^ CDE = apparent digestibility coefficients for energy; ^6^ DP = (crude protein feed x coefficient for digestible protein)/100; ^7^ DE = (energy feed × coefficient for digestible energy)/100. The ingredients used in the commercial diet were mainly fish meal (290 g kg^−1^), soybean meal (150 g kg^−1^), corn gluten (111 g kg^−1^), wheat gluten (140 g kg^−1^), pea meal (80 g kg^−1^), wheat (50 g kg^−1^), fish oil (130 g kg^−1^), soybean oil (30 g kg^−1^), antioxidant BHT, vitamin A (5000 Ul), vitamin D3 (750 Ul), and vitamin E (150 mg kg^−1^).

**Table 3:** Growth and parameters of the two experiments of *Argyrosomus regius* fed at different feeding rates and corporal analysis of *A. regius* fed at different feeding rates at the end of the trial.

			Trial A							Trial B				
			Feeding Rate (%)							Feeding Rate (%)				
	0		0.75	1.5	2.5	3.5	4.5	SEM		0	0.5	1.5	2.5	SEM
**Performance Indices**														
IW ^1^		55	52	52	53	54	54	2		140 ^a^	209 ^b^	209 ^b^	197 ^b^	9
FW ^2^		38 ^a^	57 ^b^	70 ^bc^	77 ^bc^	88 ^c^	81 ^c^	4		111 ^a^	248 ^b^	295 ^c^	339 ^c^	7.92
TGC ^3^ × 10^−3^		−0.93 ^a^	0.27 ^b^	0.91 ^c^	1.25 ^c^	1.69 ^c^	1.4 ^c^	0.15		−0.98 ^a^	0.95 ^b^	1.88 ^c^	2.74 ^d^	0.15
FI ^4^		0 ^a^	0.50 ^b^	1.05 ^c^	1.72 ^d^	2.22 ^e^	2.75 ^f^	0.10		0 ^a^	0.38 ^b^	1.03 ^c^	1.88 ^d^	0.06
DPI ^5^		0 ^a^	1.65 ^b^	3.53 ^c^	5.72 ^d^	7.25 ^e^	8.79 ^f^	0.40		0 ^a^	1.59 ^b^	4.46 ^c^	8.07 ^c^	0.24
PR ^6^		−0.61 ^a^	0.68 ^b^	1.26 ^c^	1.62 ^cd^	1.81 ^d^	1.61 ^cd^	0.11		−0.80 ^a^	1.08 ^b^	1.89 ^b^	2.33 ^b^	0.19
DEI ^7^		0 ^a^	91.5 ^b^	193.9 ^c^	311.5 ^d^	393.0 ^e^	481.7 ^f^	22.1		0 ^a^	78.4 ^b^	217.1 ^c^	395.8 ^d^	10.0
ER ^8^		−18.9 ^a^	34.8 ^b^	62.8 ^c^	77.9 ^cd^	90.8 ^d^	81.2 ^cd^	5.0		−29.9 ^a^	44.3 ^b^	92.1 ^c^	119.9 ^c^	10.4
**Carcass Composition**														
	Initial								Initial					
Moisture	80.06	82.83 ^a^	77.50 ^b^	75.52 ^c^	75.23 ^c^	74.06 ^c^	74.35 ^c^	0.40	79.35	83.54 ^a^	76.10 ^b^	74.29 ^b^	71.59 ^b^	1.09
Protein	11.91	11.76 ^a^	14.82 ^b^	15.77 ^c^	16.02 ^d^	16.30 ^e^	16.10 ^de^	0.05	13.24	11.76 ^a^	15.57 ^b^	16.24 ^b^	16.36 ^b^	0.57
Lipid	1.95	1.96 ^a^	4.21 ^b^	5.46 ^c^	5.80 ^c^	6.60 ^c^	6.24 ^c^	0.34	1.96	0.76 ^a^	3.89 ^b^	6.42 ^b^	7.69 ^b^	0.76
Ash	4.34	4.63 ^a^	3.38 ^b^	3.29 ^b^	3.09 ^b^	3.33 ^b^	3.29 ^b^	0.22	4.68	4.39 ^a^	4.03 ^b^	3.13 ^b^	3.88 ^b^	0.22
GE ^9^	3.62	3.59 ^a^	5.22 ^b^	5.94 ^c^	6.13 ^c^	6.52 ^c^	6.33 ^c^	0.14	3.94	3.11 ^a^	5.27 ^b^	6.43 ^b^	6.97 ^b^	0.40

Means of duplicate groups. Data in the same row not sharing a common superscript letter are significantly different (*p* < 0.05). SEM: pooled standard error of the mean. ^1^ IW = initial weight (g), was considered covariable to FW, TGC, digestible protein intake (DPI), protein retention (PR), digestible energy intake (DEI), and energy retention (ER); ^2^ FW = Final Weight (g); ^3^ thermal growth coefficient (TGC) = 1000 × [final weight (g) ^1/3^—initial weight (g)^1/3^]/((T°—minimum T° to feed) × days); minimum T° to feed = 12 °C; ^4^ feed intake (FI, % day^−1^) = 100 × feed consumption (g)/average biomass (g) × days; ^5^ digestible protein intake (g kg^−0.7^day^−1^); ^6^ protein retention (g kg^−0.7^day^−1^); ^7^ digestible energy intake (kJ kg^−0.82^ day^−1^); ^8^ energy retention(kJ kg^−0.82^ day^−1^); ^9^ GE: gross energy, calculated using 23.9 kJ g^−1^ proteins, 39.8 kJ g^−1^ lipids, and 17.6 kJ g^−1^ carbohydrates.

**Table 4:** Protein and energy retention key points.

**Protein Retention Key Points**				
	DPI	PR	Gross Efficiency	Net Efficiency
Maintenance	0.57	0	0	-
Max. Retention	6.0	1.81	0.30	0.33
Max. Efficiency	1.8	0.94	0.52	0.76
**Energy Retention Key Points**				
	DEI	ER	Gross Efficiency	Net Efficiency
Maintenance	25.4	0	0	-
Max. Retention	365	92.2	0.25	0.27
Max. Efficiency	93	43.5	0.47	0.64

DPI = digestible protein intake (g kg^−0.7^day^−1^); PR = protein retention (g kg^−0.7^day^−1^); DEI = digestible energy intake (kJ kg^−0.82^ day^−1^); ER = energy retention (kJ kg^−0.82^ day^−1^).

**Table 5:** Maintenance protein and energy requirements estimated for several fish species.

Species	Maintenance Protein Requirements	Maintenance Energy Requirements	T^a^ (°C)	Study
Meagre (*Argyrosomus regius*)	0.57 g DP kg^−0.7^ fish^−1^ day^−1^	25.4 kJ DE kg^−0.82^ fish^−1^ day^−1^	19	Present study
Gilthead sea bream (*Sparus aurata*)	0.86 g DP kg^−0.7^ fish^−1^ day^−1^	55.8 kJ DE kg^−0.83^ fish^−1^ day^−1^	23–24	[20]
Gilthead sea bream (*Sparus aurata*)	1.39 g DP kg^−0.7^ fish^−1^ day^−1^	59.84 kJ DE kg^−0.82^ fish^−1^ day^−1^	21–25	[19]
European sea bass (*Dicentrarchus labrax*)	0.66 g DP kg^−0.69^ fish^−1^ day^−1^	43.6 kJ DE kg^−0.79^ fish^−1^ day^−1^	19–26	[22]
European sea bass (*Dicentrarchus labrax*)	0.87 g DP kg^−0.7^ fish^−1^ day^−1^	50.9 kJ DE kg^−0.8^ fish^−1^ day^−1^	25	[23]
White grouper (*Epinephelus aeneus*)		34.05 kJ DE kg^−0.8^ fish^−1^ day^−1^	19–27	[21]
Mulloway (*Argyrosomus japonicus*)	0.47 g DP kg^−0.7^ fish^−1^ day^−1^	44.21–49.59 kJ DE kg^−0.8^ fish^−1^ day^−1^	20–26	[25]
Red drum (*Sciaenops ocellatus*)	0.5–2.2 g DP kg fish^−1^ day^−1^	58 to 97 kJ DE kg fish^−1^ day^−1^	25	[26]
Yellowtail (*Seriola quinqueradiata*)	2.7–3.1 g DP kg fish^−1^ day^−1^	62.7 kJ DE kg fish^−1^ day^−1^	22–27	[39]

## Data Availability

This article is self-explanatory and the data needed are provided allready inside the article.
